# Effect of N-Methylacetamide Concentration and Thawing Rate on Chicken Sperm Quality after Cryopreservation

**DOI:** 10.3390/ani10050824

**Published:** 2020-05-09

**Authors:** Fabio Mosca, Luisa Zaniboni, Ahmad Abdel Sayed, Nicolaia Iaffaldano, Dominga Soglia, Achille Schiavone, Silvia Cerolini

**Affiliations:** 1 Dipartimento di Medicina Veterinaria, University of Milan, via dell’Università 6, 26900 Lodi, Italy; fabio.mosca1@unimi.it (F.M.); luisa.zaniboni@unimi.it (L.Z.); ahmad.abdel@unimi.it (A.A.S.); 2 Dipartimento di Agricoltura, Ambiente e Alimenti, University of Molise, via De Sanctis, 86100 Campobasso, Italy; nicolaia@unimol.it; 3 Dipartimento di Scienze Veterinarie, University of Turin, Largo Paolo Braccini 2, 10095 Grugliasco, Torino, Italy; dominga.soglia@unito.it (D.S.); achille.schiavone@unito.it (A.S.)

**Keywords:** sperm cryopreservation, cryoprotectants, N-Methylacetamide, thawing temperature, cryodamage

## Abstract

**Simple Summary:**

The semen cryopreservation technology is still the only efficient method for the *ex situ* conservation of genetic diversity in birds. This study investigates the effect of different concentrations (6% and 9%) of the cryoprotectant N-Methylacetamide and of different thawing temperatures (at 5 °C for 100 s; 38 °C for 30 s) on chicken semen quality after cryopreservation. The cryoprotectant concentration significantly affected sperm membrane integrity, total and progressive motility after cryopreservation and this effect was dependent by the thawing temperature. The treatment that provided the best cryoprotective action and decreased the cellular cryodamage was the concomitant use of 6% N-Methylacetamide and thawing at 5 °C for 100 s. These results can contribute to improve the efficacy of the current chicken semen cryopreservation technology.

**Abstract:**

In seeking alternative cryoprotectants to glycerol for a reference chicken semen freezing procedure, the aim of the present study was to compare the effect of two concentrations of N-Methylacetamide (MA) and two thawing rates on the quality of frozen-thawed semen. Semen samples were diluted in Lake pre-freezing extender, including 0.1 M trehalose in presence of 6% or 9% MA, loaded into straws, frozen in nitrogen vapors, and stored in liquid nitrogen. The following thawing treatments were used: 5 °C for 100 s and 38 °C for 30 s. Sperm quality (cell membrane integrity, motility and kinetic parameters) was assessed before and after cryopreservation. The decrease of MA concentration from 9 to 6% improved sperm quality after freezing/thawing and this effect was dependent on thawing temperature. Decreasing the MA concentration from 9 to 6% improved the proportion of undamaged membrane, motile, and progressive motile sperm recovered after thawing at 5 °C for 100 s; in contrast, no effect of the MA concentration was observed thawing at 38 °C for 30 s. Therefore, the treatment with 6% MA and thawing at 5 °C for 100 s has given the best cryoprotective action. These results contribute to improve the efficacy of the current chicken semen cryopreservation procedures.

## 1. Introduction

Sperm cryopreservation is still the only efficient method for the *ex situ in vitro* management of genetic diversity in birds [1,2]. According to the international agreements on animal biodiversity [3] and the increase of poultry genetic resources conservation programs [4], the avian sperm cryopreservation technology urgently requires deeper investigations in order to be improved and standardized.

Semen cryopreservation involves several critical points affecting sperm integrity and function such as dilution, type of cryoprotectant and its concentration, packaging system, and freezing/thawing rate [5,6]. Furthermore, the interaction of these factors can also affect the success of sperm cryopreservation [7].

The choice of the cryoprotective agent (CPA) is certainly among the most important factors for effective poultry semen freezing protocols. Glycerol (GLY) is probably the most effective and the least toxic permeant CPA (P-CPA) for chicken spermatozoa [8] but, unfortunately, its concentration must be decreased to less than 1% prior to insemination, due to its well-recognized contraceptive effect in the hen oviduct [9,10]. To date, the P-CPAs dimethylsulphoxide (DMSO), dimethylacetamide (DMA), dimethylformamide (DMF), ethylene glycol (EG) and N-Methylacetamide (MA) have been tested for chicken semen cryopreservation [6]. Among these alternatives to GLY, MA is the most recently studied: the quality of chicken sperm cryopreserved with MA is only partially described and there is a significant inconsistency in results regarding its true ability to prevent cell freezing-induced damages [11,12,13]. Furthermore, MA displayed a negative concentration-dependent effect on fertility [14]. Consequently, the related fertilization rate remains highly variable: in chickens, the fertility after artificial insemination with frozen/thawed semen cryopreserved with MA ranged from 0 [15] to 100 % [16].

In addition to the type of CPA, thawing rate is a critical phase for the success of semen cryopreservation [17]. Lower sperm quality was reported using DMA as CPA and a thawing rate of 37°C for 30 s [18] compared to 5°C for 120 s in presence of EG [19]. In contrast, Miranda et al. [12] reported no differences comparing the effect of two thawing temperatures (5 vs 37 °C) on sperm motility of chicken semen cryopreserved with MA. 

In seeking alternative CPAs to GLY for a reference chicken semen freezing procedure, the aim of the present study was to compare the effect of two concentrations of MA and two thawing rates on the quality of frozen-thawed semen. 

## 2. Materials and Methods 

### 2.1. Bird Management and Semen Collection

Thirty adult Hi-Line White male fowl (*Gallus gallus domesticus*) were housed at 28 weeks of age in individual cages and kept at 20° C and 14L:10D photoperiod at the Poultry Unit, Animal Production Centre, University of Milan (Lodi, Italy). Birds were fed *ad libitum* with standard commercial chicken breeder diet (2800 kcal ME/kg, 15% CP) and drinking water. Bird handling was in accordance with the principles presented in Guidelines for the Care and Use of Agricultural Animals in Research and Teaching [20]. After 2-week of semen collection training period, all males were routinely collected twice a week from October to November. Semen was collected according to the technique initially described by Burrows and Quinn [21]. Each day of collection, males were randomly divided in three different groups (ten birds/group) and all ejaculates collected within one group were pooled into one semen sample. Pooled semen samples obtained in different days were always created with different ejaculates to reduce the effect of the bird.

The Animal Welfare Committee of the University of Milan evaluated and approved the experimental protocol (OPBA_94_2017).

### 2.2. Semen Processing for Cryopreservation

Semen volume was recorded in graduated tubes and sperm concentration was measured after 1:200 dilution in 0.9% NaCl using a calibrated photometer (IMV, L’Aigle, France) at a wavelength of 535 nm [22]. Each pooled semen sample was diluted to a concentration of 1.5 × 10^9^ sperm/mL using a Lake pre-freezing modified extender (LPF-T) [23]. The diluted semen was immediately cooled and kept at 4° C for 30 minutes. During this incubation, semen samples were transferred to the laboratory for further quality assessment and freezing processing. The quality assessment included sperm membrane integrity (MI), total motility (TM), progressive motility (PM), and kinetic parameters. Sperm MI was measured using the dual fluorescent staining SYBR14/propidium iodide procedure (LIVE/DEAD Sperm Viability Kit, Molecular Probes, Invitrogen), as described by Rosato and Iaffaldano [24] with minor modifications. In brief, the incubations were done at room temperature and the 7.1 diluent [25] was used. Assessment of 200 spermatozoa was made in duplicate aliquots for every sample and evaluated microscopically at 1000 × magnification using a Zeiss (Axioskop 40- AxioCamICc 1) microscope and FITC filter fluorescence. Sperm TM (%), PM (%) and motion parameters were assayed using a computer-aided sperm analysis system coupled with a phase contrast microscope (Nikon Eclipse model 50i; negative contrast) employing the Sperm Class Analyzer (SCA) software (version 4.0, Microptic S.L., Barcelona, Spain). Pooled semen samples were further diluted in refrigerated 0.9% NaCl to a sperm concentration of 100×10^6^/mL and incubated for 20 minutes at room temperature. Then, 10 μL semen was placed on a Makler counting chamber (Sefi Medical Instruments, Haifa, Israel) and evaluated under the microscope at 100× magnification and room temperature. A minimum of three fields and 500 sperm tracks were analyzed. The sperm motion parameters included values on sperm velocity, velocity ratios, the amplitude of lateral head displacement [ALH, (μm)] and the beat cross frequency [BCF, (Hz)]. Sperm velocity parameters (μm/s) were: curvilinear velocity (VCL), straight-line velocity (VSL), and average path velocity (VAP). The velocity ratios (%) were: linearity of the curvilinear path [LIN (VSL/VCL)], straightness of the average path [STR (VSL/VAP)] and wobbling [WOB (VAP/VCL)] [26]. After sperm quality analyses, pooled semen samples were splitted into two aliquots and diluted in LPF-T in order to provide each sample with the following treatment: 1) LPF-T added with 18% MA to reach the final 1×10^9^ sperm/mL and 6% MA concentrations (M-6); 2) LPF-T added with 27% MA to reach the final 1×10^9^ sperm/mL and 9% MA concentrations (M-9). After equilibration at 5°C for 1 min, semen was loaded into 0.25 mL French straws (IMV Technologies, France) and frozen for 10 min over a nitrogen bath at 3 cm of height [27]. Straws were transferred into a cryotank and stored for at least 7 days. Twelve pooled semen samples (12 replicates/treatment) were processed and a total of 48 straws were stored per treatment. Straws were thawed in water bath according to the following treatment: a) at 5 °C for 100 s; b) at 38 °C for 30 s. Sperm MI, TM, PM, and motion parameters were recorded immediately after thawing as previously described for fresh semen samples.

The thawing temperature gradient inside straws (n = 2 per treatment) was measured with a thermometer fitted with a probe resistant to freezing (80PK-1K, Fluke-51/RS, Fluke Corporation, USA). Before freezing, the probe was introduced into a straw and the temperatures were constantly recorded during thawing.

### 2.3. Statistical Analysis

Analysis of variance for repeated measures was applied by the MIXED procedure of SAS [28]. The variables studied were the sperm quality parameters measured before and after cryopreservation; the statistical model included the CPA concentration (6% vs 9%), the thawing temperature (5 °C for 100 s vs 38 °C for 30 s), the time of sampling (before vs after cryopreservation) and the relative interactions as fixed effects and the pooled semen samples as random effect. LSMeans were compared with the Student’s t test. The recovery rates (%) of sperm MI, TM, and PM after cryopreservation were calculated as follows: [(mean on thawed semen × 100)/mean on fresh semen]. Analysis of variance on the recovery variables was applied by the GLM procedure of SAS [28], and the CPA concentration (6% vs. 9%), the thawing temperature (5 °C per 100 s vs 38 °C per 30 s), and the relative interaction were the sources of variation included in the model. LSMeans were compared with the Student’s t test. The arcsine transformation was used to normalize all percentage data before statistical analysis. Results are presented as LSMean ± SEM.

## 3. Results

### 3.1. Semen Quality

The mean volume and sperm concentration recorded in fresh ejaculates were 0.18 ± 0.02 mL and 3.70 ± 0.44 × 109 sperm/mL respectively.

The results of the analysis of variance on semen quality parameters are shown in Table 1. All the sources of variation and their interactions significantly affected sperm MI, TM, PM, and the kinetic parameter VSL and VAP. The thawing temperature, the freezing/thawing process and the relative interaction significantly affected the kinetic parameters VCL, LIN, and WOB, whereas none of the treatments and interactions significantly affected STR, ALH, and BCF (Table 1). 

The mean values of sperm quality parameters recorded before and after cryopreservation in semen samples processed according to different MA concentrations and thawing rates are reported in Table 2. An overall significant decrease in sperm quality occurred after the freezing-thawing process and the mean proportion of undamaged membrane, motile, and progressive motile sperm recorded in fresh and thawed semen was respectively 90 vs. 32%, 88 vs. 31%, and 23 vs. 5%. The decrease of MA concentration from 9% to 6% significantly improved the sperm quality parameters after freezing/thawing and this effect was dependent on thawing temperature and present only at 5 °C (Table 2). Therefore, the treatment 6 % MA * 5 °C for 100 s performed the best cryoprotective action and significantly improved MI, TM, PM, VSL and VAP in thawed semen (Table 2). 

Moreover, the thawing temperature significantly affected the whole quality of cryopreserved chicken semen, irrespective of the cryoprotectant concentration (significant interaction TR*T in Table 1). In fact, the slower thawing rate, 5 °C for 100 s, also significantly improved the kinetic parameters VCL (44 vs 33 µm/s), LIN (40 vs 33%) and WOB (63 vs 55 %) in comparison to the faster thawing rate, 38 °C for 30 s.

The recovery rate of undamaged membrane (*p* < 0.05), motile (*p* < 0.05) and progressive motile (*p* < 0.05) sperm was significantly affected by the CPA concentration, the thawing rate, and the relative interaction.

The mean recovery values recorded in semen samples frozen with 9 and 6% MA and thawed at 5 °C for 100 s and 38 °C for 30 s are reported in Table 3. The effect of the CPA concentration on the recovery values of undamaged membrane, motile and progressively motile sperm changed according to the thawing rate. Decreasing the CPA concentration from 9 to 6%, the proportion of undamaged membrane, motile, and progressively motile sperm recovered after thawing at 5 °C for 100 s significantly improved; in contrast, the CPA concentration did not affect the proportion of recovered sperm variables if semen was thawed at 38 °C for 30 s (Table 3). 

### 3.2. Thawing Rates

**Figure 1:**
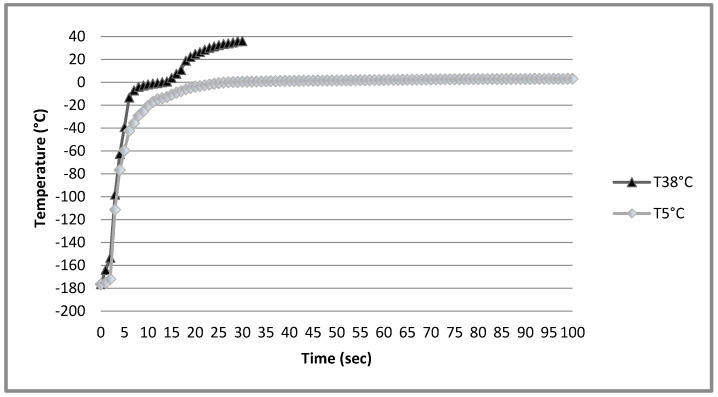
Change in temperature of chicken semen inside straws during thawing procedure according to different thawing rates.

As represented in Figure 1, each temperature/time ratio corresponds to a different temperature gradient during thawing. The temperature inside straws thawed at 5 °C/100 s increased to 0 °C within a 25 s term and then never increased to above 3 °C. In straws thawed at 38°/30 s, the same temperature of 0 °C was reached after about only 10 s and then the temperature increased to above 30 °C within a further 10 s.

## 4. Discussion

To date, a variety of P-CPAs such as DMSO, DMA, DMF, EG, and MA were tested as alternatives to GLY to achieve success of chicken semen cryopreservation [6,12,15].

MA [H3C-C(O)-N(H)-CH3] is yielded by replacement of the sulfinyl group [-S(O)-] in DMSO with an amide group [-C(O)-N(H)-]. Because the amide group is naturally involved in bioprocess, MA presented a lower cytotoxicity on cryopreserved human cells compared to DMSO [23]. Furthermore, when compared to DMA, MA imposed a lower toxic effect on the somites in cultured rat embryos [29]. According to the low *in vitro* toxicity and to the inconsistency of the results reported in the current literature on its cryoprotective function on chicken sperm, the study on MA to standardize the procedure to freeze chicken semen is still actual.

Regardless of the presence of toxic atomic groups, each P-CPA shows a cytotoxic effect, related to the high molar concentrations used in the cryopreservation extenders, acting before freezing and after thawing [30]. Thus, the lowest concentration of any CPA required for an effective cryopreservation procedure should be determined. In the present study, the cryoprotectant effect of two concentrations of MA on sperm *in vitro* quality was compared in chicken semen packaged in straws, according to the FAO cryopreservation guidelines [31]. The MA concentration of 9% was selected according to several reports on fertilization trials using frozen/thawed chicken semen [11,13,14,16]. However, in those studies, an *in vitro* assessment of semen quality were only partially [11,13,14] or totally absent [16] and the average fertility ranged from 0 [13] to 100 % [16]. The 6% MA was selected with the goal to decrease the P-CPA concentration in our semen freezing procedure. To our knowledge, a complete *in vitro* assessment of both cell membrane integrity and function of chicken semen frozen/thawed in presence of MA at different concentrations is reported for the first time. The results showed that 6% MA, compared to 9%, performed a best protective action during cryopreservation of chicken sperm, improving cell MI, TM, PM, and the related recovery rates. On the contrary, Pranay Kumar et al. [13] reported similar cell membrane integrity proportion in presence of both 6 and 9 % MA. Furthermore, decreasing MA concentration from 9 to 6% improved some sperm quality parameters indicative of high quality motion, such as VCL that represents a well-recognized fertility-related parameter in roosters [32].

In addition to CPA concentration, the success of sperm cryopreservation procedure is also related to other factors such as the thawing conditions [17]. It is generally accepted that thawing rates should mimic freezing rates [33] and, according to our rapid freezing rate protocol [27], thawing at 38 °C for 30 s should be a more suitable procedure. In fact, according to the curves reported in Figure 1, thawing at 38 °C is twice faster compared to thawing at 5 °C. Surprisingly, the thawing rate of 5 °C for 100 s increased the cryoprotective ability of MA on chicken semen resulting in a general improvement of *in vitro* sperm quality. Many of these sperm quality parameters improved by the 5 °C thawing conditions are considered predictive of the fertilizing potential of cryopreserved semen: VAP has been largely investigated in mammalian species and related to high semen fertility [34], while VCL was significantly higher in human sperm which were able to perform penetration in *in vitro* assay compared to those failing [35]. Probably, the true effect of the slower thawing rate on sperm quality is related to the overall lower temperature that might have better preserved avian sperm membrane, in agreement with its high sensitivity to lipoperoxidation compared to mammalian species [36]. However, the present results are in disagreement with those of Miranda et al. [12] who reported no differences comparing the effect of two thawing temperatures (5 °C vs 37 °C) on sperm motility of chicken semen cryopreserved with MA. Furthermore, Iaffaldano et al. [17] reported that thawing conditions of 50 °C for 10 s were more efficient than 4 °C for 5 min for turkey sperm provided with DMSO before freezing.

Another interesting point emerging from the present study is the significant interaction between P-CPA concentration and thawing rate affecting the *in vitro* cryopreservation success. At the thawing condition of 5 °C, the lower MA concentration of 6% improved several sperm quality parameters. On the contrary, the higher MA concentration of 9% played the same cryoprotective function on chicken semen irrespective of thawing temperature. According to post-thaw semen quality, the combination of lower P-CPA concentration/lower thawing temperature represented the best option for chicken semen provided with MA before freezing. The sperm cell membrane integrity, motility and progressive motility in semen samples frozen/thawed according to the 6%/5 °C treatment were 51%, 52% and 11% respectively. Using a similar cryopreservation method in the presence of MA 9%, Lee et al. [11] reported lower sperm cell membrane integrity (34%) in White Leghorn and Korean Oge chickens, while Miranda et al. [12] reported lower sperm motility (32 %) but higher progressive motility (16%) in commercial strains of chicken. Furthermore, Miranda et al. [12] confirmed that the 9% MA plays the same cryoprotective action on chicken semen irrespective of thawing rate. Pranay Kumar et al. [13] showed lower cell membrane integrity proportion (35%) irrespective of CPA concentration for freezing of chicken semen in presence of 6 and 9% MA. Finally, the combination of 6% MA and the thawing at 5 °C increased the recovery of undamaged membrane, motile and progressively motile sperm, decreasing the sensitivity of fresh semen to the freezing/thawing process.

## 5. Conclusions

The present study provided evidence that decreasing the P-CPA MA from 9 to 6% improves the *in vitro* quality of chicken semen after cryopreservation. Moreover, the combination of the low 6% MA concentration with the low thawing temperature, corresponding to 5° for 100 s, caused a further improvement in chicken semen quality after cryopreservation. The relevance to confirm these positive *in vitro* results with *in vivo* fertility data is highlighted. These results contribute to the identification of a chicken semen cryopreservation procedure effective in the reduction of sperm cryodamage that could be successfully implemented in an avian genetic resource semen cryobank. 

## Figures and Tables

**Table 1:** Results of Analysis of Variance: *p* values of the sources of variation cryoprotectant concentration (C), thawing rate (TR), time of sampling (T) and the relative interactions included in the statistical General Linear Model applied to study chicken sperm quality before and after cryopreservation.

Sperm Parameters ^1^	C	TR	T	C*TR	C*T	TR*T	C*TR*T
MI	<0.001	<0.001	<0.001	<0.05	<0.001	<0.001	<0.05
TM	<0.001	<0.001	<0.001	<0.05	<0.05	<0.001	<0.05
PM	<0.05	<0.001	<0.001	<0.05	<0.05	<0.001	<0.05
VCL	<0.001	<0.001	<0.001	ns	ns	<0.001	ns
VSL	<0.05	<0.001	<0.001	<0.05	<0.05	<0.001	<0.05
VAP	<0.001	<0.001	<0.001	<0.05	<0.001	<0.001	<0.05
LIN	ns	<0.001	<0.001	ns	ns	<0.001	ns
STR	ns	ns	ns	ns	ns	ns	ns
WOB	ns	<0.001	<0.001	ns	ns	<0.001	ns
ALH	ns	ns	ns	ns	ns	ns	ns
BCF	ns	ns	ns	ns	ns	ns	ns

^1^ MI membrane integrity: percentage undamaged membrane spermatozoa; TM total motility: percentage motile spermatozoa; PM progressive motility: spermatozoa swim forward fast in a straight line; VCL: curvilinear velocity; VSL: straight-line velocity; VAP: average path velocity; ALH: amplitude of lateral head displacement; BCF: beat cross frequency; LIN: linearity = (VSL/VCL × 100); STR: straightness = (VSL/VAP × 100); WOB: wobble = (VAP/VCL × 100).

**Table 2:** Sperm quality parameters (LSMeans ± SEM) measured in fresh and frozen/thawed semen cryopreserved using different concentration of N-Methylacetamide and different thawing temperatures.

Sperm Parameters ^1^	Fresh Semen	Frozen/Thawed Semen				SEM
		6% 5 °C 100s	6% 38 °C 30s	9% 5 °C 100s	9% 38 °C 30s	
Membrane integrity (%)	89.9 ^A^	50.7 ^B^	22.8 ^D^	36.6 ^C^	20.5 ^D^	1.8
Motility (%)	87.7 ^A^	52.3 ^B^	20.0 ^D^	35.5 ^C^	18.1 ^D^	2.3
Progressive motility (%)	23.1 ^A^	11.2 ^B^	2.2 ^D^	4.9 ^C^	1.2 ^D^	0.9
VCL (µm/s)	55.5	49.1	34.9	39.3	31.7	1.6
VSL (µm/s)	23.6 ^A^	20.9 ^B^	11.9 ^D^	15.3 ^C^	10.6 ^D^	0.8
VAP (µm/s)	36.8 ^A^	32.0 ^B^	19.2 ^D^	23.7 ^C^	17.5 ^D^	1.1
LIN (%)	42.6	42.4	33.8	38.9	33.7	1.3
STR (%)	64.2	64.9	61.6	64.3	61.0	1.2
WOB (%)	66.3	65.0	54.7	60.3	55.2	1.1
ALH (µm)	2.8	3.1	2.9	3.1	2.9	0.1
BCF (Hz)	7.5	7.2	6.5	7.3	6.7	0.4

^1^ Membrane integrity: percentage undamaged membrane spermatozoa; Motility: percentage motile spermatozoa; Progressive motility: spermatozoa swim forward fast in a straight line; VCL: curvilinear velocity; VSL:straight-line velocity; VAP: average path velocity; ALH:amplitude of lateral head displacement; BCF: beat cross frequency; LIN: linearity=(VSL/VCL x 100); STR: straightness= (VSL/VAP x 100); WOB: wobble= (VAP/VCL x 100). ^A, B, C, D^; SEM: standard error of mean. Values within a row with no common superscripts differ significantly at *p* < 0.001 among treatments.

**Table 3:** Recovery (LSMeans ± SEM) of undamaged membrane, motile and progressively motile sperm after cryopreservation with different N-Methylacetamide concentration-thawing rate combinations.

Sperm Variables ^1^	Recovery Rates (%)				SEM
	6% 5 °C 100 s	6% 38 °C 30 s	9% 5 °C 100s	9% 38 °C 30s	
Membrane integrity	56.5 ^A^	25.3 ^C^	40.7 ^B^	22.9 ^C^	2.7
Motility	59.5 ^A^	22.6 ^C^	40.6 ^B^	20.7 ^C^	3.1
Progressive motility	49.6 ^A^	9.1 ^C^	21.9 ^B^	5.3 ^C^	4.3

^1^ Membrane integrity: percentage of undamaged membrane spermatozoa; Motility: percentage motile spermatozoa; Progressive motility: spermatozoa swim forward fast in a straight line ^A, B, C^; SEM: standard error of mean. Different superscripts show a significant difference among treatments within row at *p* < 0.001.
